# Label-Free LC-MS Profiling of Skeletal Muscle Reveals Heart-Type Fatty Acid Binding Protein as a Candidate Biomarker of Aerobic Capacity 

**DOI:** 10.3390/proteomes1030290

**Published:** 2013-12-18

**Authors:** Zulezwan A. Malik, James N. Cobley, James P. Morton, Graeme L. Close, Ben J. Edwards, Lauren G. Koch, Steven L. Britton, Jatin G. Burniston

**Affiliations:** 1 Research Institute for Sport and Exercise Sciences, Liverpool John Moores University, Liverpool, L3 3AF, UK; E-Mails: z.ab-malik@2011.ljmu.ac.uk (Z.A.M.); j.cobley@abertay.ac.uk (J.N.C.); j.p.morton@ljmu.ac.uk (J.P.M.); g.l.close@ljmu.ac.uk (G.L.C.); b.j.edwards@ljmu.ac.uk (B.J.E.); 2 Department of Anesthesiology, University of Michigan, Ann Arbor, MI 48109-2200, USA; E-Mails: lgkoch@med.umich.edu (L.G.K.); brittons@umich.edu (S.L.B.)

**Keywords:** aerobic capacity, animal selection model, exercise training, heart-type fatty acid binding protein, FABPH, Fabp3, human vastus lateralis, selective reaction monitoring

## Abstract

Two-dimensional gel electrophoresis provides robust comparative analysis of skeletal muscle, but this technique is laborious and limited by its inability to resolve all proteins. In contrast, orthogonal separation by SDS-PAGE and reverse-phase liquid chromatography (RPLC) coupled to mass spectrometry (MS) affords deep mining of the muscle proteome, but differential analysis between samples is challenging due to the greater level of fractionation and the complexities of quantifying proteins based on the abundances of their tryptic peptides. Here we report simple, semi-automated and time efficient (*i.e*., 3 h per sample) proteome profiling of skeletal muscle by 1-dimensional RPLC electrospray ionisation tandem MS. Solei were analysed from rats (n = 5, in each group) bred as either high- or low-capacity runners (HCR and LCR, respectively) that exhibited a 6.4-fold difference (1,625 ± 112 m *vs*. 252 ± 43 m, *p* < 0.0001) in running capacity during a standardized treadmill test. Soluble muscle proteins were extracted, digested with trypsin and individual biological replicates (50 ng of tryptic peptides) subjected to LC-MS profiling. Proteins were identified by triplicate LC-MS/MS analysis of a pooled sample of each biological replicate. Differential expression profiling was performed on relative abundances (RA) of parent ions, which spanned three orders of magnitude. In total, 207 proteins were analysed, which encompassed almost all enzymes of the major metabolic pathways in skeletal muscle. The most abundant protein detected was type I myosin heavy chain (RA = 5,843 ± 897) and the least abundant protein detected was heat shock 70 kDa protein (RA = 2 ± 0.5). Sixteen proteins were significantly (*p* < 0.05) more abundant in HCR muscle and hierarchal clustering of the profiling data highlighted two protein subgroups, which encompassed proteins associated with either the respiratory chain or fatty acid oxidation. Heart-type fatty acid binding protein (FABPH) was 1.54-fold (*p* = 0.0064) more abundant in HCR than LCR soleus. This discovery was verified using selective reaction monitoring (SRM) of the y5 ion (551.21 *m/z*) of the doubly-charged peptide SLGVGFATR (454.19 *m/z*) of residues 23–31 of FABPH. SRM was conducted on technical replicates of each biological sample and exhibited a coefficient of variation of 20%. The abundance of FABPH measured by SRM was 2.84-fold greater (*p* = 0.0095) in HCR muscle. In addition, SRM of FABPH was performed in vastus lateralis samples of young and elderly humans with different habitual activity levels (collected during a previous study) finding FABPH abundance was 2.23-fold greater (*p* = 0.0396) in endurance-trained individuals regardless of differences in age. In summary, our findings in HCR/LCR rats provide protein-level confirmation for earlier transcriptome profiling work and show LC-MS is a viable means of profiling the abundance of almost all major metabolic enzymes of skeletal muscle in a highly parallel manner. Moreover, our approach is relatively more time efficient than techniques relying on orthogonal separations, and we demonstrate LC-MS profiling of the HCR/LCR selection model was able to highlight biomarkers that also exhibit differences in trained and untrained human muscle.

## 1. Introduction

Proteome profiling has proved to be a pragmatic means of discovering novel information and identifying new avenues of research. In the field of skeletal muscle proteomics, differential expression analysis has been used to investigate human proteome responses to exercise [1], ageing [2], bed-rest [3] and obesity [4]. In addition, proteomic techniques have been applied to a wide variety of experimental models, including denervation [5] and electrical stimulation [6]. These works have used two-dimensional gel electrophoresis to resolve proteins, which are then identified from mass spectrometry of in-gel digests. This approach offers robust comparative analysis of protein species, which represent different post-translational states of a protein [7]. However, 2D electrophoresis requires a significant level of skill, is laborious and has a number of technical limitations, including difficulties in resolving proteins at the extremes of the mass and pH scales, and a limited dynamic range. 

Efforts have been made to perform differential analyses using high-performance liquid chromatography (LC) coupled online with mass spectrometry (MS). This approach promises largely automated analysis but currently it is necessary to conduct profiling on tryptic peptides rather than intact proteins, which brings a substantive increment in sample complexity and severs the connection linking site-specific post-translational modifications with functional protein species. Nonetheless, useful information regarding changes in protein abundance can be collected, but the density of peptide profiling data places a formidable burden on separation and mass spectrometry technologies. In muscle proteomics, the time efficiency of orthogonal gel and liquid separations has been investigated [8]. The combination of SDS-PAGE with LC-ESI-MS/MS, known as GeLC-MS/MS [9] is of particular utility in skeletal muscle protein identification. However, spectral counting (e.g., [10]) or peptide labelling techniques (e.g., [1]) are necessary in order to perform differential profiling using GeLC-MS/MS. GeLC-MS/MS also requires substantial (~30 h) machine time for each biological replicate, due to the sequential analysis of individual fractions of each sample. 

Here we address the question of whether separation of soluble muscle proteins using simple one-dimensional liquid chromatography provides useful information regarding muscle phenotype. Skeletal muscle is ideal substrate for such label-free LC-MS profiling because the abundance of metabolic enzymes and myofibrillar proteins that underpin muscle function is high and spans a relatively narrow dynamic range. Phenotyping of muscle is typically based on the relative distribution of myosin heavy chain (MyHC) isoforms. However, such classification ignores the fact that myofibres can exhibit broadly different metabolic properties yet have identical MyHC content (see [11]). Indeed, while changes in the metabolic and contractile properties of skeletal muscle often co-occur (e.g., during ageing [2,12]), in most instances muscle metabolism is more labile than myofibrillar structure. In this regard, metabolic profiling is likely to be a more sensitive and discriminative means of describing muscle than myofibre phenotype. 

Traditionally, the capacity of metabolic pathways is inferred from the maximal activities of key rate-limiting enzymes, including citrate synthase (CS; tricarboxylic acid cycle), phosphofructokinase (PFK; glycolysis), 3-hydroxyacyl-CoA dehydrogenase (β-HAD; β-oxidation), or the concentrations of marker proteins such as cytochrome c (respiratory chain) [13]. However, such assays require relatively large amounts of raw material and must be performed independently. Proteomic techniques provide more comprehensive and parallel analysis, and this may aid the discovery of biomarkers of disease processes. For example, it is conceivable, that an enzyme not typically recognised as being rate-limiting under normal circumstances could nonetheless influence flux through the pathway under specific conditions. In addition, many well characterised metabolic enzymes are known to also contribute to cellular processes other than metabolism. For example, glyceraldehyde-3-phosphate dehydrogenase (GAPDH), which is typically the most abundant glycolytic enzyme in skeletal muscle, also plays a key role in nitrosylation of nuclear proteins [14]. Here we report LC-MS profiling of slow-twitch soleus muscle from two strains of rats, which have been artificially selected based on either high- or low-running capacity (HCR or LCR, respectively) [15]. Previously we reported proteomic analysis of hearts [16] from this model, but to date skeletal muscle has not been investigated using proteomic techniques. Based on previous literature (e.g., [17]) we did not expect soleus fibre type to be different between HCR and LCR but it is expected that HCR and LCR soleus will exhibit differences in metabolic profile, which makes it an ideal substrate for this work.

## 2. Experimental

The inception of HCR-LCR strains from a founder population of genetically heterogeneous N:NIH rats has been described in detail [15]. Ten male HCR/LCR rats (*n* = 5, in each group) from generation 25 (12–13 weeks old) were imported from the University of Michigan. The transfer of animals to the UK and subsequent procedures were conducted under the British Home Office Animals (Scientific Procedures) Act 1986 and according to UK Home Office Guidelines. Rats were housed in a conventional facility and the environmental conditions controlled at 20 ± 2 °C, 45%–50% relative humidity with a 12 h light (06:00–18:00 h) and dark cycle. Food and water were available *ad libitum* during a 14-day acclimatization period. After an overnight fast (~10 h), animals were asphyxiated with CO_2_ and killed by cervical dislocation. Skeletal muscles and other organs were isolated and cleaned of fat and connective tissue before being weighed and frozen in liquid nitrogen.

Soleus muscles were pulverised in liquid nitrogen then homogenised on ice in 8 volumes of 1% Triton X-100, 50 mM Tris pH 7.4 containing Complete™ protease inhibitor (Roche Diagnostics, Lewes, UK). Samples were incubated on ice for 15 min then centrifuged at 1,000 rpm, 4 °C for 5 min. Supernates were precipitated in acetone and resuspended in Lysis Buffer: 7 M urea, 2 M thiourea, 4% (w/v) CHAPS, 30 mM Tris, containing Complete™ protease inhibitor (Roche Diagnostics, Lewes, UK). After clearing by centrifugation (12,000 *g*, 4 °C for 45 min) protein concentrations were measured using the Bradford assay (Sigma, Poole, Dorset, UK) and each sample adjusted to 5 μg μL^−1^. Aliquots containing 100 μg protein were precipitated in 5 volumes of acetone for 1 h at −20 °C. Pellets were resuspended in 0.1% (w/v) Rapigest SF (Waters; Milford, MA, USA) in 50 mM ammonium bicarbonate and incubated at 80 °C for 15 min. DTT was added (final concentration 1 mM) and incubated at 60 °C for 15 min followed by incubation whilst protected from light in the presence of 5 mM iodoacetamide at 4 °C. Sequencing grade trypsin (Promega; Madison, WI, USA) was added at a protein ratio of 1:50 and digestion allowed to proceed at 37 °C overnight. Digestion was terminated by the addition of 2 μL concentrated TFA and peptide solutions were cleared by centrifugation at 13,000 *g* for 5 min. 

Label-free liquid chromatography-mass spectrometry (LC-MS) analysis was performed using a quadrupole-high capacity ion-trap (HCT Ultra ETD II; Bruker Daltonics, Bremen, Germany) coupled online via an electrospray ionisation source to a nano-flow HPLC system (Ultimate 3000; Dionex, Sunnyvale, CA, USA). Tryptic digests (0.8 μg/μL) were diluted 1:10 with aqueous 0.1% formic acid (FA) and 5 μL loaded via a Zorbax 300SB C_18_ 5 μm, 5 × 300 μm pre-column (Agilent Technologies Ltd.). Peptides were separated using a Zorbax 300SB C_18_ 3.5 μm, 15 cm × 75 μm analytical reverse phase column (Agilent Technologies Ltd.) at a flow rate of 300 nL min^−1^ using a non-linear gradient rising to 40% acetonitrile 0.1% FA over 160 min. Mass spectra for LC-MS profiling were recorded between 200 *m/z* and 2,500 *m/z* using Standard Enhanced mode [8,100 (*m/z*)/s]. In addition, equivalent data-dependent tandem mass spectrometry (MS/MS) spectra were collected from triplicate analysis of a pooled standard comprising each HCR and LCR sample. MS/MS spectra of collision-induced dissociation fragment ions were recorded for the 5 most abundant precursors from each survey scan (350 *m/z* to 1,600 *m/z*).

Both MS and MS/MS spectra were aligned using Progenesis LC-MS software (Nonlinear Dynamics, Newcastle, UK). Prominent ion features (mean ± SD per chromatogram: 455 ± 42) were used as vectors to warp each dataset to a common reference chromatogram. An analysis window of 15 min – 145 min and 200 *m/z* – 1,800 *m/z* was selected, which encompassed a total of 32,824 features with charge states of +2, +3 or +4. Log transformed MS data were normalised by inter-sample abundance ratio and used to investigate differences in expression between LCR and HCR groups by one-way analysis of variance. MS/MS spectra (16, 872 queries) were exported in Mascot generic format and searched against the Swiss-Prot database (2011.6) restricted to ‘Rattus’ (7617 sequences) using a locally implemented Mascot server (version 2.2.03). The enzyme specificity was trypsin allowing 1 missed cleavage, carbamidomethyl modification of cysteine (fixed), deamidation of asparagine and glutamine (variable), oxidation of methionine (variable) and an *m/z* error of ± 0.5 Da. The Mascot output (xml format), restricted to non-homologous protein identifications was recombined with MS profile data in Progenesis LC-MS. Peptide features with MOWSE scores <10 (MudPIT scoring) were excluded and potential conflicts in peptide assignments were resolved manually.

Functional annotation was conducted using the Database for Annotation, Visualization and Integrated Discovery (DAVID; [18]). Over-representation of gene ontology (GO) classes: cellular component (CC), biological process (BP) and molecular function (MF) was investigated. Association of proteins with pathways of the Kyoto Encyclopedia of Genes and Genomes (KEGG; [19]) was also assessed.

Selective reaction monitoring (SRM) was used to verify the difference in heart-type fatty acid binding protein (FABPH) abundance detected by LC-MS profiling. FABPH exhibited the greatest fold difference between HCR and LCR muscles, and SRM of serum FABPH levels has previously been reported [20]. SRM was performed using similar instrument settings described for LC-MS profiling but samples were separated using a linear chromatographic gradient rising to 40% acetonitrile 0.1% FA in 15 min, rather than 160 min used during the initial profiling experiment. Samples were analysed in duplicate and in a randomised order. A single transition 454–551 was monitored, which represents the y5 ion (551.21 *m/z*) of the doubly charged peptide SLGVGFATR (2 + 454.19 *m/z*) of residues 23–31 of FABPH. This peptide is identical in rat and human so we also performed the SRM assay on human muscle collected during a separate experiment, reported in [21]. Biopsy samples of vastus lateralis of young or elderly individuals that had either high or low levels of habitual physical activity, were processed and analysed equivalently to the HCR/LCR soleus muscle. Statistical analysis was conducted by 2-way analysis of variance (age versus activity level). 

## 3. Results

### 3.1. Physical and Physiological Characteristics of HCR and LCR Rats

**Figure 1:**
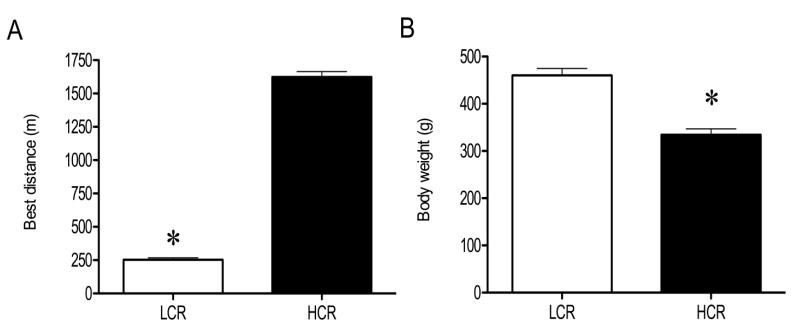
Physical characteristics of high-(HCR) and low-capacity runner (LCR) rats. Furthest distance (m) completed during a standardised treadmill test of LCR and HCR rats at approximately 10 weeks of age (**A**). Body weight of LCR and HCR rats prior to tissue harvesting at approximately 14 weeks of age (**B**). Data are expressed as Mean ± SD (*n* = 5, in each group), * *p* < 0.05.

The body weight of LCR (460 ± 40 g) was 1.38-fold greater (*p* < 0.0001) than HCR (334 ± 36 g) and there was a 6.44-fold difference (*p* < 0.0001) difference in running capacity (1,625 ± 112 m *vs*. 252 ± 43 m) between HCR and LCR strains (Figure 1).

### 3.2. LC-MS Profiling of Soluble Muscle Proteins

**Figure 2:**
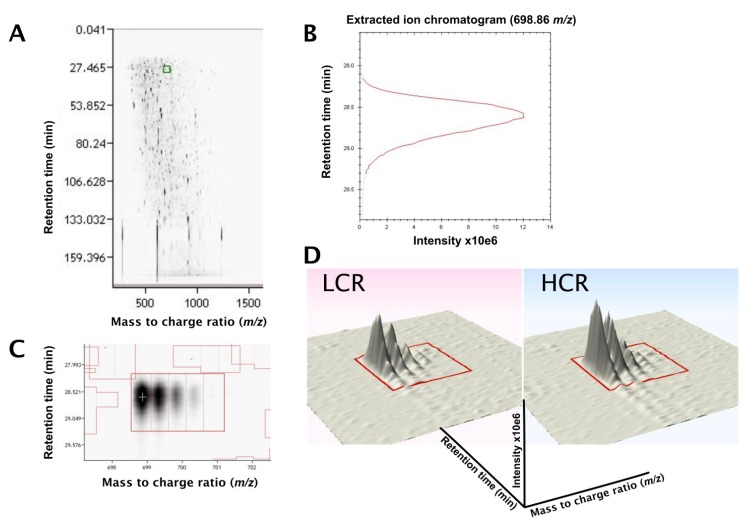
Label-free LC-MS profiling. LC-MS spectrum (**A**) showing the separation of tryptic peptides by reversed-phase liquid chromatography retention time (min) and mass-to-charge ratio (*m/z*). Inset highlights the area of selection of an extracted ion chromatogram (**B**) of peptide 698.86 *m/z*. Demarcation of the isotope envelope of peptide 698.86 *m/z* (**C**) and 3-dimensional representation of the peptide in representative LCR and HCR samples (**D**).

In total 207 protein entries (2,999 peptides) mapped to features in progenesis LC-MS and were used for quantitative analysis. Figure 2 illustrates the data analysis process and a spreadsheet (Table S1) detailing the entire list of Mascot protein identifications and normalised protein abundance data is available. Based on normalised ion intensities the most abundant proteins included type I myosin heavy chain (MYH7), carbonic anhydrase, skeletal muscle alpha actin and creatine kinase. The least abundant proteins detected were heat shock protein 70 kDa protein 4, enoyl-CoA delta isomerase 2, and nuclear pore membrane protein 210. Normalised relative abundances (RA) of proteins analysed in the current work spanned 3 orders of magnitude from 2 (heat shock 70 kDa protein, HSP74) to 5,843 ± 897 (type I myosin heavy chain, MYH7). As expected, the relative abundance of enzymes that contribute to the first phase of glycolysis (e.g., phosphoglucomutase 1, RA = 95 ± 9) was approximately half that of enzymes involved in the second phase (e.g., phosphoglycerate mutase 2, RA = 174 ± 23). Consistent with the slow-twitch phenotype of the soleus, the ratio of lactate dehydrogenase A (RA = 122 ± 12) to lactate dehydrogenase B (RA = 234 ± 65) was approximately 1:2, and the ratio of fast troponin I (RA = 9 ± 2) to slow troponin I (RA = 77 ± 15) was approximately 1:9. Although the soleus typically contains approximately 20% type IIa fibres, proteotypic peptides of myosin-2 (MYH2) were not detected, therefore, the ratio of type I and IIa fibres cannot be reported.

**Figure 3:**
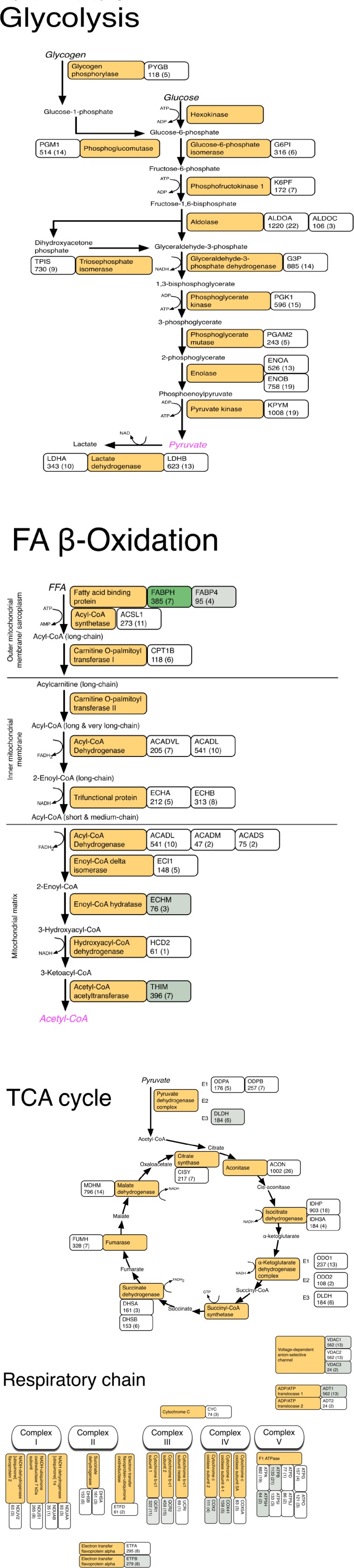
LC-MS profiling of muscle metabolic enzymes. The metabolic pathways of glycolysis, fatty acid β-oxidation and the tricarboxylic acid cycle are redrawn from the Kyoto Encyclopedia of genes and genomes (KEGG). For clarity the respiratory chain is not shown in its entirety, instead only subunits detected by LC-MS profiling are highlighted. Orange boxes display the common name of each enzyme, the adjacent box details the UniProt Rattus ID identified by LC-MS and the number of peptides attached to this sequence. Numbers in parentheses represent the number of detected peptides that were unique to that protein and subsequently used for differential expression profiling. A ‘heat map’ coloring system is used to display proteins that were significantly more abundant in HCR (green) or LCR (red) groups.

Functional annotation of the non-redundant protein list revealed the top-ranked biological process was generation of precursor metabolites and energy (53 proteins) and the most abundant cellular component was mitochondrion (95 proteins). The main molecular functions were: nucleotide binding (66 proteins), and metal ion binding (60 proteins). With the exception of 4 proteins (carnitine *O*-palmitoyl transferase II, hexokinase, pyruvate dehydrogenase E2 component and succinyl-CoA synthetase) each enzyme of the major pathways of fatty acid oxidation, glycolysis and the tricarboxylic acid cycle was quantified (*i.e*., 2 or more unique peptides) in the current LC-MS profiling experiment (Figure 3). 

**Figure 4:**
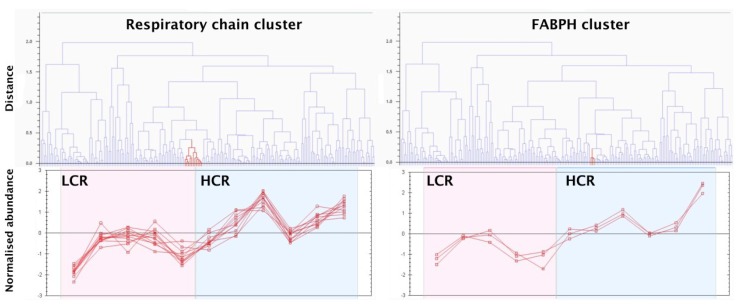
Hierarchal clustering of differentially expressed proteins. Hierarchal clustering highlighted 2 prominent groupings within LC-MS profiling data. Eleven proteins associated with the respiratory chain, that were differentially expressed in HCR and LCR soleus (see Table 1) shared patterns of variation between individual animals. Similarly, 3 of the differentially expressed proteins associated with fatty acid metabolism were also clustered based on their shared variability.

Differential analysis of HCR and LCR samples highlighted 16 proteins (Table 1) that were more abundant (*p* < 0.05) in HCR soleus. No proteins were found to be significantly more abundant in LCR muscle. Proteins more abundant in HCR muscle were associated with aerobic metabolism and hierarchal clustering highlighted two prominent groups of proteins (respiratory chain and fatty acid metabolism) that shared patterns of expression across the biological replicates (Figure 4).

**Table 1:** Differences in protein abundance between LCR and HCR soleus muscle. Description and Database ID relate to the protein name and accession number identified from Mascot searches of the UniProt Rattus database. Protein abundance relative differences (fold difference) in HCR compared to LCR are reported for proteins exhibiting significant (*p* < 0.05) differences in abundance at a FDR of <10%.

Description	Database ID	MOWSE (peptides)	Relative Abundance LCR	Relative Abundance HCR	Fold Diff.	*p* value
**Respiratory chain**						
**Complex III**						
Cytochrome b-c1 complex subunit 1	QCR1	322 (11)	109.87 ± 8.8	121.26 ± 5	1.10	0.0274
Cytochrome b-c1 complex subunit 2	QCR2	459 (15)	166.77 ± 10.6	182.25 ± 8.7	1.09	0.0290
**Complex IV**						
Cytochrome c oxidase subunit 2	COX2	111 (4)	50.93 ± 4.2	58.16 ± 5.9	1.14	0.0458
Cytochrome c oxidase subunit 4 isoform 1	COX41	159 (5)	68.78 ± 5	79.39 ± 7.3	1.15	0.0206
**Complex V**						
ATP synthase subunit beta	ATPB	1185 (21)	740.15 ± 66.1	843.71 ± 69.4	1.14	0.0326
ATP synthase subunit d	ATP5H	64 (2)	40.96 ± 2.8	47.47 ± 3.4	1.16	0.0081
ADP/ATP translocase 1	ADT1	562 (10)	457.55 ± 45.4	528.89 ± 40.5	1.16	0.0232
Voltage-dependent anion-selective channel protein 1	VDAC1	412 (12)	197.73 ± 13.1	221.68 ± 11.3	1.12	0.0111
Voltage-dependent anion selective channel protein 3	VDAC3	53 (4)	45.82 ± 3.7	52.94 ± 3.9	1.16	0.0137
Electron transfer flavoprotein subunit beta	ETFB	279 (8)	113.23 ± 8.6	128.03 ± 10.4	1.13	1.13
**Fatty acid transport**						
Fatty acid-binding protein, heart	FABPH	349 (7)	328.72 ± 62.3	504.97 ± 116.5	1.54	0.0064
Fatty acid-binding protein, adipocyte	FABP4	95 (4)	48.06 ± 5.2	55.47 ± 5.7	1.15	0.0426
Methylmalonate-semialdehyde dehydrogenase	MMSA	115 (5)	55.59 ± 2	58.63 ± 5	1.05	0.0235
Dihydrolipoyl dehydrogenase	DLDH	184 (6)	57.83 ± 3.8	66.78 ± 4.9	1.15	0.0077
3-Ketoacyl-CoA thiolase	THIM	396 (7)	89.21 ± 10.6	111.88 ± 11.4	1.25	0.0071
Enoyl-CoA hydratase	ECHM	76 (3)	21.76 ± 2.2	25.97 ± 3.1	1.19	0.0245

### 3.3. SRM Verification of FABPH

**Figure 5:**
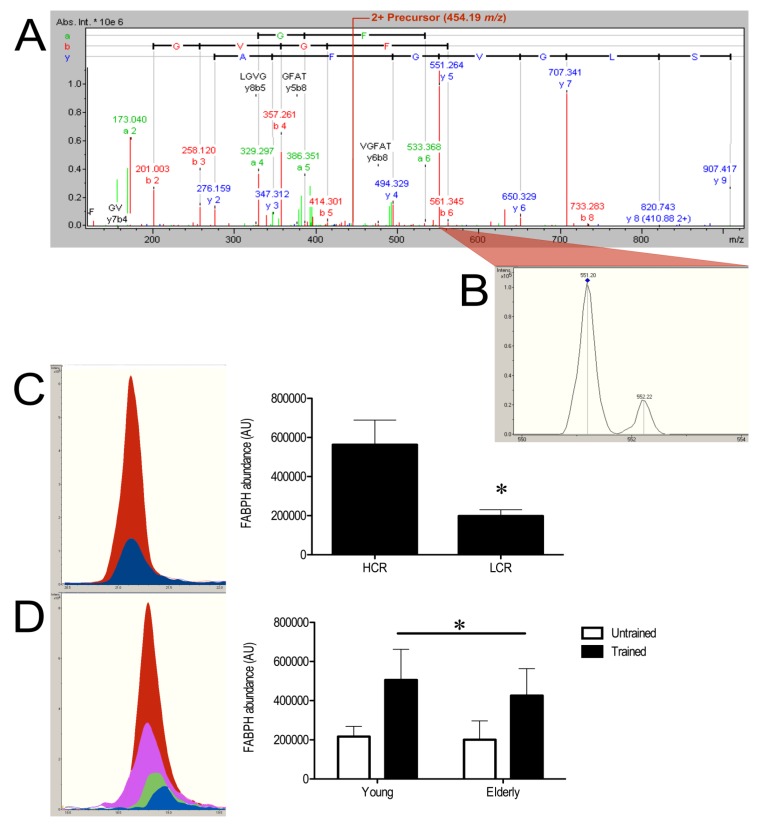
Selective reaction monitoring of FABPH. Annotated MS/MS spectra of the doubly-charged precursor, 454.19 *m/z* (**A**) of residues 23–31 (SLGVGFATR) of FABPH and (**B**) monitoring of the selected fragment ion (551.21 *m/z*). Selective reaction monitoring chromatograms of the 454.19 *m/z* to 551.21 *m/z* transition in HCR and LCR soleus (**C**) and human vastus lateralis (**D**). In C, the red shaded area represents a HCR sample and the blue area represents a LCR sample. In D, data from a young trained individual (red) is overlaid by data from elderly trained (pink), elderly untrained (green) and young sedentary (blue) individuals. The histograms represent the ion abundance of the areas under the chromatogram curves for the respective groups. Data are presented as means ± SD (*n* = 5, in each group), * *p* < 0.05.

LC-MS profiling found FABPH exhibited the greatest difference (1.54-fold, *p* = 0.0064) in abundance between HCR and LCR muscle. SRM verified this finding and measured a 2.84-fold greater (*p* = 0.0095) abundance in HCR soleus (Figure 5a). The reproducibility of the SRM method was assessed using technical replicates of HCR and LCR samples. The assay exhibited no significant systematic bias in retention time or area under the curve of the ion chromatogram, which was used to estimate abundance. The average retention time was 21 ± 0.2 min (coefficient of variation 0.4%) and the coefficient of variation of measures of FABPH abundance was 20%. SRM of FABPH in human muscle found training status significantly (*p* = 0.0396) effected FABPH abundance. Endurance-trained humans had 2.23-fold greater levels of FABPH compared to untrained individuals (Figure 5b), whereas FABPH content was not significantly affected by age.

## 4. Discussion

The current work reports a time- and sample-efficient method of profiling the expression of the major metabolic enzymes in skeletal muscle. Using a simple LC-MS approach we surveyed more than 200 proteins, including almost all enzymes of the major metabolic pathways (*i.e*., glycolysis, fatty acid β-oxidation, the tricarboxylic acid cycle and some components of the respiratory chain; Figure 3). Recently [10], spectral counting was successfully combined with GeLC-MS/MS to perform differential analysis of 438 proteins in human skeletal muscle. However, that relatively broad coverage of the muscle proteome was achieved at the expense of substantially greater machine time and sample usage. Hussey *et al*. [10] separated 60 μg aliquots of muscle homogenate in to 20 molecular weight fractions, which were analysed by LC-MS/MS over 90 min periods. This equates to roughly 1,800 min machine time per sample (biological replicate). In the current work, approximately 50 ng of tryptic peptides from each biological replicate was resolved by LC-MS of 180 min duration. This represents a 10-fold improvement in time efficiency and a greater than 100-fold increase in sample efficiency; albeit at the expense of a 2-fold decrease in the number of proteins detected. We believe the current approach represents a useful compromise that may lead to more widespread application of LC-MS profiling as a semi-automated and efficient means of phenotyping skeletal muscle.

Here, we applied LC-MS profiling to muscle of HCR and LCR rats that exhibit differences in exercise capacity, cardiometabolic disease risk and life expectancy [22]. Deterioration in the metabolic flexibility of skeletal muscle is associated with insulin resistance, which is a cardinal feature of human metabolic syndrome, and also accompanies natural ageing. Metabolic dysfunction and insensitivity to insulin are also evident as correlated traits of selection on low running capacity. For example, in the basal (fasted) state LCR muscle has lesser uptake and oxidation of fatty acids and, when stimulated by insulin, glucose uptake of LCR hindlimb muscles is significantly less than HCR [23]. Transcriptome profiling [17] reports enrichment of gene-sets, including oxidative phosphorylation, fatty acid metabolism and PPAR signalling, correlate with the differences in running activity of LCR and HCR animals. However, such differences between HCR and LCR are generally more pronounced in predominantly fast- compared to slow-twitch muscles. The proportion of type IIa MyHC (42 ± 16% versus 22 ± 14%) was significantly greater in HCR gastrocnemius used for transcriptome analysis, which may have contributed to some of the differences observed in metabolic markers. To obviate effects due to myofibre profile, we performed proteomic analysis of slow-twitch soleus muscle that has consistent myofibre proportions (~80% type I, ~20% type IIa) in HCR and LCR rats [17]. 

LC-MS profiling detected 16 significant differences between HCR and LCR soleus muscles. Consistent with the selection paradigm and earlier reported [17] differences in soleus mitochondrial content, the majority of proteins were more abundant in HCR muscle (Table 1) and were associated with the mitochondrial respiratory chain. Hierarchal clustering of the LC-MS profiling data highlighted 2 principal groups of differentially expressed proteins (Figure 4), which either encompassed components of the respiratory chain or were associated with fatty acid metabolism. This outcome supports the earlier [17] gene-set enrichment analysis of HCR/LCR gastrocnemius transcriptomes and provides protein-level confirmation for some of the transcript data. Specifically, HCR soleus had greater abundance of cytochrome b-c1 complex subunit 1 and cytochrome c oxidase subunit 4, isoform 1, which were included in the oxidative phosphorylation gene-set enriched in HCR gastrocnemius muscle. In addition, 3-ketoacyl-CoA thiolase (THIM) and enoyl-CoA hydratase (ECHM) included in the HCR gastrocnemius gene-set “Fatty acid oxidation” were also more abundant in HCR soleus (Table 1). In the current work, THIM and ECHM abundances correlated closely with FABPH (Figure 4), which was listed separately under “PPAR signalling” by gene-set enrichment analysis. Thus two separate and unbiased profiling approaches have come to highly consistent conclusions regarding prominent networks (and biomarkers thereof) underpinning innate differences in muscle aerobic capacity.

On average, the aerobic capacity of HCR hindlimb muscles is ~80% greater than LCR and this difference is underpinned by both a larger yield and greater efficiency of mitochondria isolated from HCR animals [24]. Cytochrome c oxidase subunit 1 is commonly used as a mitochondrial biomarker because it is encoded from the mitochondrial genome and is the main catalytic subunit of the terminal complex (Complex IV) of the electron transport chain. Consistent with earlier findings (e.g., [25,26]) the abundance of COX1 is significantly greater in mitochondria isolated from HCR hindlimb muscle [24]. However, differences in the abundance of protein biomarkers of other complexes of the electron transport chain were not detected [24], which led the authors to conclude the greater aerobic capacity of HCR is in part due to differences in flux control of the respiratory chain. COX1 was not detected by LC-MS profiling but we report significant differences in the abundance of several other proteins associated with the respiratory chain, including subunit 1 of Complex III and adenine nucleotide transferase (ANT), which were not significantly different when investigated by western blotting of mitochondria isolated from the entire hindlimb [24]. In addition, we report greater abundance in HCR soleus of mitochondrial encoded COX subunit 2 and nuclear encoded COX subunit 4, as well as 2 subunits of F1-ATP synthase. These data support earlier findings [26] reporting significantly greater COXI in HCR soleus, along with greater abundance of cytochrome b-c1 complex subunit 2, peroxisome proliferator-activated receptor gamma (PPARγ), PPARγ co-activator 1 alpha, and F1-ATPase. An elevated abundance of muscle ATP synthase subunits is commonly reported in response to exercise training [27] and our current data add to evidence that ATP synthase may represent a candidate biomarker of muscle aerobic capacity. Interestingly, rate-limiting enzymes such as citrate synthase, which are traditionally used to assay muscle metabolism were not differentially expressed between HCR and LCR. 

A decline in maximal aerobic capacity occurs with advancing age and, consistent with data from humans, the life expectancy of HCR and LCR rats can be predicted by their maximum oxygen uptake (VO2max) during adulthood [22]. Maximum lifespan is similar in HCR and LCR populations but the median age of death is ~24 months for LCR and 30 months for HCR, which equates to a 28% difference in life expectancy [22]. While LCR exhibit comparatively greater deterioration of cardiac function during ageing, impairments in muscle aerobic metabolism may also contribute to the relatively poor life expectancy of animals selected for low running capacity. The interaction between ageing and aerobic capacity is complex and likely each is underpinned by multigenic mechanisms. For example, Bell *et al*. [28] reports a protein interaction network of 2,338 nodes constructed from 175 human homologs of genes that modify lifespan when mutated in model organisms such as yeast and Caenorhabditis elegans. This ‘longevity interactome’ was then restricted to genes that also exhibit significant differences in expression between muscle of young and elderly individuals to highlight a core subnetwork of 325 age-associated proteins [28]. Amongst those core proteins tightly associated with ageing were COX41, FABPH and ATP5H, which were found to be differentially expressed between HCR and LCR soleus muscle (Table 1). 

FABPH exhibited the most prominent difference between HCR and LCR muscles, and we used selective reaction monitoring (SRM) to verify this initial discovery. SRM assays afford greater selectivity and sensitivity than LC-MS profiling and can often be performed more rapidly. During LC-MS profiling relative quantification is achieved by monitoring the intensity of parent-ion masses but this may be susceptible to interference from unrelated peptides of similar mass-to-charge ratio. In contrast, SRM analysis monitors the intensity of a known product-ion mass during fragmentation of a selected parent ion (known as a transition) and this additional level of filtering affords greater selectivity and sensitivity. SRM analysis of FABPH in rat serum has previously been reported [20] and we report an identical parent/fragment ion transition can be used in rat skeletal muscle with good reliability (*i.e*., 20% coefficient of variation). Our method development was also aided by use of the same MS platform for both LC-MS profiling and SRM [29], and we were able to shorten analysis time per sample by a factor of 10. SRM confirmed our discovery by LC-MS profiling that FABPH is significantly more abundant in HCR muscle (Figure 5C). FABPH binds long-chain fatty acids in the sarcoplasm and the abundance of this protein is tightly associated with the capacity of muscle to take up fatty acids [30]. Thus the lesser FABPH content of LCR muscle is likely to be a key factor contributing to the lesser uptake of FA in LCR muscle (e.g., [23]) under basal conditions. In addition FABPH is also known to have reciprocal effects on the utilisation of glucose and long-chain fatty acids during exercise [31]. Therefore the lesser abundance of FABPH in LCR muscle may also play a role in the relative poor running capacity of these animals. Paradoxically, FABPH is less abundant in muscle of normal-weight individuals compared to obese and morbidly obese patients [4], which may be expected to have relatively poorer exercise capacity. This phenomenon is likely to be connected with the elevated storage of intramuscular triacylglycerides, which occurs in both obese individuals and endurance-trained athletes [32]. The link between FABPH and the longevity interactome [28] is consistent with proteomic analysis in rats. Proteomic analysis of FABPH in muscle of young and elderly rats [33] reports significantly greater abundance of FABPH in elderly gastrocnemius but this could be due to the loss of fast-twitch fibres and relatively greater proportion of slow-twitch fibres in gastrocnemius of old rats [34]. In contrast, we found no significant difference in FABPH abundance between normal-weight young and elderly people (Figure 5D). Whereas, endurance-trained individuals had a 2.23-fold greater abundance of FABPH, which suggests age-associated declines in physical activity or training status (*i.e*., muscle aerobic capacity) may have greater influence on FABPH abundance than age per se.

## 5. Conclusions

In summary, we report time efficient and automated differential analysis of the top 200 abundant proteins in skeletal muscle. Further strengths of this work include sample efficiency and the breadth and parallel nature of the analysis, which eliminates variability introduced by performing numerous enzyme activity assays in series. Using a model of artificial selection we highlight prominent biomarkers of aerobic capacity and show LC-MS profiling represents a viable and efficient method of phenotyping muscle based on the relative abundance of metabolic enzymes. Moreover, these data can be efficiently verified using SRM. Wider application of such techniques may lead to deeper understanding of muscle physiology and patho-physiology. In the future, use of higher resolution instrumentation may enable yet more comprehensive and rapid surveying of abundant muscle proteins and perhaps include signalling proteins that regulate these pathways.

## Supplementary Materials

Supplementary File 1
